# Real-world efficacy and prognostic factor analysis of tislelizumab versus sintilimab in advanced non-small cell lung cancer

**DOI:** 10.1186/s12885-026-16576-0

**Published:** 2026-07-22

**Authors:** Quanyi Liu, Xiaotong Zhu, Yuxuan Xie, Yun Gu, Lei Shen

**Affiliations:** 1 Department of Pharmacy, The First Affiliated Hospital of Dali University, Dali, China; 2 Department of Pharmacology, School of Pharmacy, Dali University, Dali, China

**Keywords:** Tislelizumab, Sintilimab, Non-small cell lung cancer, Clinical efficacy, Safety, Influencing factors

## Abstract

**Background:**

Tislelizumab and Sintilimab are commonly used immunotherapy drugs in clinical practice. Only a portion of advanced NSCLC patients achieve sustained remission and long-term survival through ICI immunotherapy, while others experience disease progression post-treatment. Selecting appropriate clinical therapies and predicting treatment efficacy remain significant challenges in clinical practice. By examining the clinical efficacy and safety of tislelizumab and sintilimab combined with chemotherapy in advanced NSCLC patients within real-world settings, and analyzing prognostic factors, this study aims to provide reference for clinical treatment decisions and prognosis assessment.

**Materials and methods:**

A retrospective analysis of clinical data from 212 patients with advanced NSCLC treated with the combination of tislelizumab or sintilimab plus chemotherapy in The First Affiliated Hospital of Dali University between March 1, 2022, and December 31, 2024. Patients were divided into the tislelizumab group (*n* = 124) and the sintilimab group (*n* = 88) based on the administered drug. Objective response rate (ORR), disease control rate (DCR), progression-free survival (PFS), and overall survival (OS) were evaluated in both groups. Kaplan-Meier methods were used to plot PFS and OS survival curves. Univariate analysis was performed using the Log-rank test, and Cox regression models were employed to identify independent prognostic factors for PFS and OS.

**Results:**

The median progression-free survival (mPFS) in the tislelizumab group and the sintilimab group was 14.0 months (95% CI: 11.79–16.21) and 7.0 months (95% CI: 4.31–9.69), respectively (*P* = 0.005). The difference in mPFS between the two groups was statistically significant. The median overall survival (mOS) was 27.0 months (95% CI: 20.03–33.97) and 24.0 months (95% CI: 13.85–34.15) (*P* = 0.113), with no statistically significant difference in mOS between groups. ORR was 39.52% versus 18.18% (*P* < 0.001), showing a statistically significant difference; DCR was 66.13% versus 70.54% (*P* = 0.506), with no statistically significant difference. After adjustment for confounders, multivariable Cox regression revealed no significant differences between the two agents for PFS (HR = 1.128, 95% CI 0.596–2.133, *P* = 0.712) or OS (HR = 0.664, 95% CI 0.378–1.165, *P* = 0.153). ADR occurrence showed a borderline association with improved OS (HR = 0.569, *P* = 0.093), but was not statistically significant. ALI, PNI, anti-angiogenic combination, and treatment line were not independent prognostic factors (all *P* > 0.05).

**Conclusion:**

In real-world clinical settings, tislelizumab combined with chemotherapy may bring potential improvements in objective response rate and progression-free survival in the short term for patients with advanced NSCLC. Nevertheless, sintilimab and tislelizumab exert comparable long-term survival benefits when used in combination with chemotherapy. After adjusting for confounding factors via multivariable regression analysis, no significant survival differences were confirmed between the two treatment regimens. Further prospective clinical studies with longer follow-up are required to verify the above conclusions.

## Introduction

Lung cancer ranks among the most prevalent and deadly malignant tumors globally, posing a severe threat to human health. Non-small cell lung cancer (NSCLC) is the most common clinical sub type, accounting for approximately 80% to 85% of all lung cancers [1, 2]. Most patients are diagnosed at an advanced stage, resulting in poor clinical prognosis. Immune checkpoint inhibitors (ICIs) have been widely adopted in clinical practice, improving five-year survival rates and establishing themselves as first and second-line treatments for advanced NSCLC, thereby reshaping the therapeutic landscape for this disease [3, 4]. Currently, over ten immunotherapy drugs for advanced NSCLC are approved in China, including sintilimab, tislelizumab, atezolizumab, and ipilimumab [5]. However, real-world reports indicate that only 15%-30% of advanced NSCLC patients achieve sustained response and long-term survival with ICI therapy, while others experience disease progression post-treatment. Selecting appropriate clinical therapies and predicting their efficacy remain significant challenges in clinical practice [6, 7]. This study focuses on two domestically produced PD-1 inhibitors that are covered by medical insurance, highly accessible, and widely accepted in clinical practice. By comparing the clinical efficacy and safety of sintilimab and tislelizumab combined with chemotherapy in patients with advanced NSCLC, and analyzing prognostic factors, this research aims to provide a reference for clinical treatment and prognosis assessment.

## Materials and methods

### Patients and study design

A retrospective analysis was conducted on the clinical data of eligible patients with advanced NSCLC who received ICIs combined with chemotherapy in our Hospital from March 1, 2022, to December 31, 2024. All participants were consecutively enrolled according to unified predefined inclusion and exclusion criteria, without subjective case selection. A total of 212 qualified patients were finally enrolled and divided into the tislelizumab group (*n* = 124) and the sintilimab group (*n* = 88) according to different ICI regimens.

Inclusion Criteria: (1) Age > 18 years; (2) Pathologically confirmed unresectable locally advanced or metastatic NSCLC; (3) Received at least 2 cycles of ICI (Tislelizumab or Sintilimab) plus chemotherapy; (4) Eastern Cooperative Oncology Group (ECOG) performance status ≤ 2; (5) Completion of at least one modified Response Evaluation Criteria in Solid Tumors (mRECIST) imaging assessment during treatment; (6) Complete blood count and biochemical test results within one week prior to initiating ICI plus chemotherapy; (7) Good compliance, regular follow-up visits, and willingness to participate in follow-up.

Exclusion criteria: (1) No evaluable lesions; (2) Concurrent infection or autoimmune disease; (3) Severe organ dysfunction (e.g., cardiac, cerebral, renal, hepatic); (4) Incomplete or missing clinical records; (5) Missing imaging or laboratory results during follow-up.

### Treatment regimen

All patients received either sintilimab or tislelizumab combined with chemotherapy.Sintilimab group: Sintilimab 200 mg (D1), administered as a 30-60-minute intravenous infusion every 21 days (Q3W) until disease progression or intolerable toxicity. Tislelizumab group: Tislelizumab 200 mg (D1), with the first infusion administered over no less than 60 min and subsequent infusions over no less than 30 min, every 21 days (Q3W) until disease progression or intolerable toxicity. Chemotherapy regimens include 5 options: pemetrexed + platinum, gemcitabine + platinum, paclitaxel + platinum, albumin-bound paclitaxel + platinum, docetaxel + platinum. ICIs and chemotherapy drugs are administered once every 3 weeks, continuing until disease progression (PD) or intolerable adverse reactions occur.

### Outcome evaluation criteria

Clinical response is assessed according to mRECIST criteria, including complete response (CR), partial response (PR), stable disease (SD), and progressive disease (PD). Short-term efficacy metrics include objective response rate (ORR) and disease control rate (DCR), where treatment response rate = CR rate + PR rate. Disease control rate = CR+ PR + SD.Long-term efficacy metrics include progression-free survival (PFS) and overall survival (OS). PFS refers to the time from initiation of ICI treatment until PD is confirmed; OS denotes the time from initiation of immune checkpoint inhibitor plus chemotherapy until death from any cause or the last follow-up visit.

### Data collection

Patient baseline and case data were collected via the electronic medical record system, including gender, age, height, weight, smoking history, comorbidities (metabolic disorders, immunodeficiency, diabetes, hypertension, etc.), tumor stage, treatment drugs, number of treatment lines, complete blood count, treatment regimen, treatment cycle, dosage, concomitant medications, and adverse reaction occurrence. Based on baseline data and laboratory indicators, the Prognostic Nutritional Index (PNI) and Advanced Lung Cancer Inflammatory Index (ALI) were calculated for each patient: PNI = Albumin (g/L) + 5×Absolute Lymphocyte Count(×10^9/L) [8]; ALI = Body Mass Index (kg/m²)×Albumin (g/L) /(Absolute Neutrophil Count / Absolute Lymphocyte Count).

### Follow-up

All patients were followed up regularly through clinical visits and telephone interviews. The follow-up period ended on June 30, 2025. Progression-free survival (PFS) was defined as the time from treatment initiation to radiological progression or death from any cause, whichever occurred first. Overall survival (OS) was defined as the time from treatment initiation to death from any cause. In survival analysis, patients who had not experienced an endpoint event (disease progression or death) were censored at the date of their last follow-up. Patients who were lost to follow-up or who received non-protocol anticancer therapy were also censored.

### Statistical analysis

Data entry was performed using Excel 2013 software, and analysis was conducted using SPSS 27.0 and GraphPad Prism 9.5 statistical software. Quantitative data are expressed as mean ± standard deviation. Comparisons between groups were performed using the independent samples t-test. Qualitative data are presented as counts and percentages (%). Comparisons between groups were performed using the chi-square test or Fisher’s exact probability test. Kaplan-Meier methods were used to construct PFS and OS curves, with log-rank tests performed. Results are presented as median values with 95% confidence intervals (CI). Univariate analysis of long-term efficacy (PFS, OS) was performed using the log-rank test. Independent prognostic factors for PFS and OS were evaluated using Cox regression models, with results expressed as hazard ratios (HR) and 95% CIs. Statistical significance was defined as *P* < 0.05.

## Results

### Comparison of general characteristics between the two groups

The comparison of general characteristics between the two groups showed no statistically significant differences (*P* > 0.05), the baseline characteristics of the two groups were generally similar. Results are shown in Table 1.

**Table 1:** Comparison of General Characteristics Between the Two Groups of Patients [Cases (%)]

Total evaluated		Sintilimab(*n* = 88)	Tislelizumab (*n* = 124)	χ^2^/T/Z	*P* value
Gender				0.085	0.77
	female	17(19.32)	22(17.74)		
	male	71(80.68)	102(82.26)		
Age				1.902	0.168
	๤60	26(29.55)	48(38.71)		
	≧ 60	62(70.45)	76(61.29)		
Smoking status				0.267	0.605
	Yes	57(64.77)	76(61.29)		
	NO	31(35.23)	48(38.71)		
Histological Type				0.558	0.455
	Adenocarcinoma	32(36.36)	39(31.45)		
	Squamous Cell Carcinoma	56(63.64)	85(68.55)		
Past Medical History				3.202	0.074
	Yes	39(44.32)	40(32.26)		
	NO	49(55.68)	84(67.74)		
Tumor staging				0.141	0.708
	III	32(36.36)	42(33.87)		
	IV	56(63.64)	82(66.13)		
Combined with anti-angiogenic therapy				3.137	0.077
	Yes	12(13.64)	29(23.39)		
	NO	76(86.36)	95(76.61)		
Treatment line				3.622	0.057
	1st	81(92.05)	103(83.06)		
	2nd and above	7(7.95)	21(16.94)		
ADR				2.106	0.147
	Yes	21(23.86)	41(33.06)		
	NO	67(76.14)	83(66.94)		
ALI		(13.24,46.55)	(13.87,43.62)	0.195	0.845
PNI		44.61 ± 6.27	43.43 ± 6.58	1.316	0.189

Data are presented as mean ± SD for continuous variables and *n* (%) for categorical variables. *P* values were calculated using the independent t-test for continuous variables and the chi-square test or Fisher’s exact test for categorical variables. All tests were two-sided, and *P* < 0.05 was considered statistically significant. Baseline indicators with borderline inter-group differences were adjusted in multivariate Cox regression analysis

### Comparison of treatment efficacy between the two patient groups

#### Comparison of short-term efficacy

Patients in the sintilimab plus chemotherapy group demonstrated lower ORR and DCR compared to the tislelizumab group.The difference in ORR between the two groups was statistically significant (*P* < 0.05), whereas the differences in DCR were not statistically significant (*P* > 0.05). Results are shown in Table 2.

**Table 2:** Comparison of Short-Term Efficacy Between the Two Groups [n(%)]

Groups	*n*	Clinical Outcomes				ORR	DCR
		CR	PR	SD	PD		
Sintilimab	88	0(0)	16(18.18)	46(52.27)	26(29.55)	16(18.18)	62(70.45)
Tislelizumab	124	3(2.42)	46(37.10)	33(26.61)	42(33.87)	49(39.52)	82(66.13)
χ^2^						11.02	0.442
P value						๤0.001	0.506

#### Comparison of long-term efficacy

The median progression-free survival (mPFS) in the sintilimab plus chemotherapy group was 7 months (95% CI:4.31–9.69), while the mPFS in the tislelizumab group was 14 months (95% CI:11.79–16.21). The difference in mPFS between the two groups was statistically significant (*P* = 0.005).

**Fig. 1:**
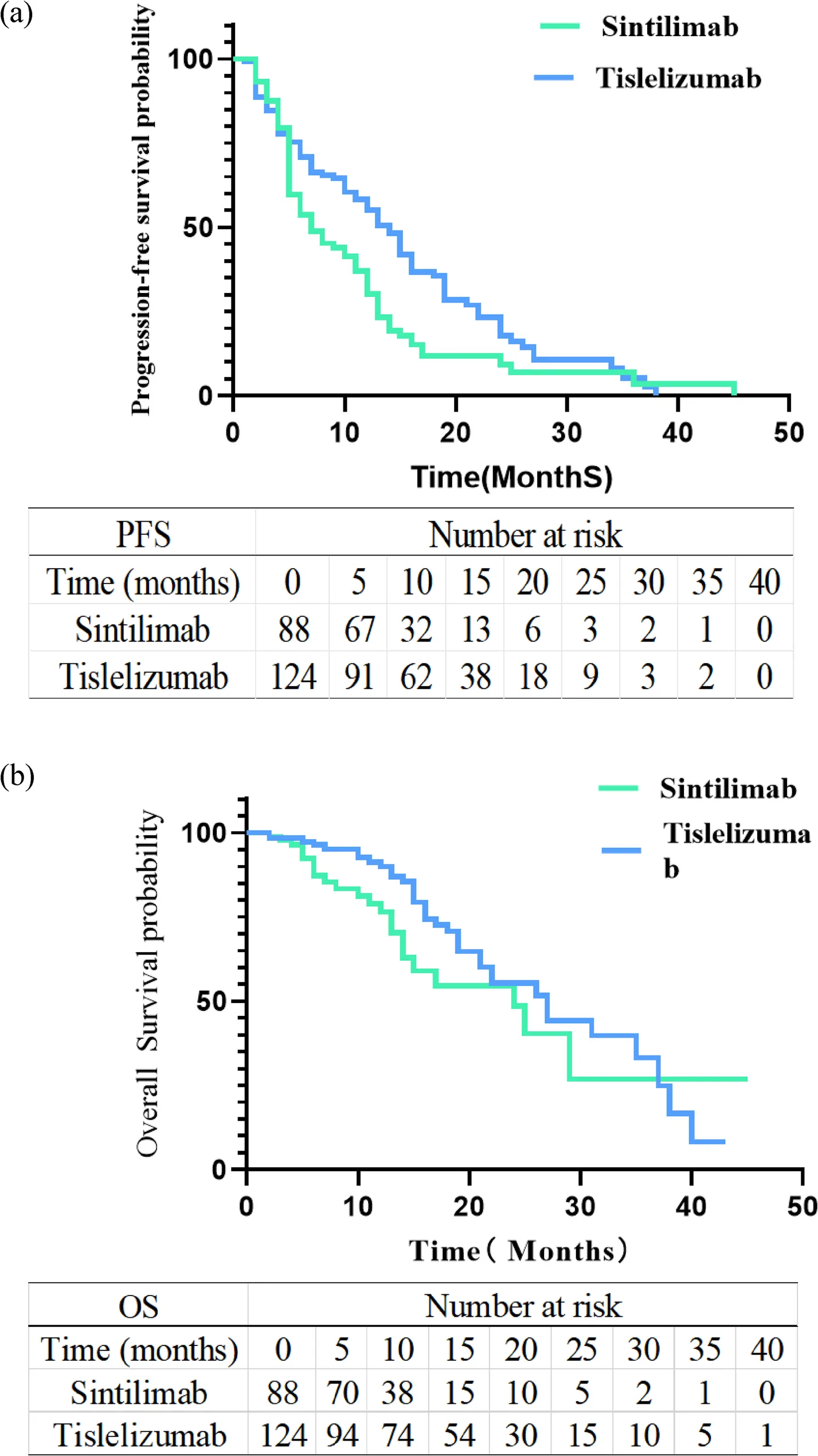
PFS (**a**) and OS (**b**) survival curves for the two groups

The mOS in the sintilimab plus chemotherapy group was 24 months (95% CI: 13.85–34.15), while the mOS in the tislelizumab plus chemotherapy group was 27 months (95% CI: 20.03–33.97). The difference in mOS between the two groups was not statistically significant (*P* = 0.113). The PFS and OS survival curves for both groups are shown in Fig. 1.

### Analysis of factors influencing long-term efficacy of ICIs

#### Univariate analysis results

Log-rank tests for PFS and OS revealed that age, number of treatment lines, PNI, and ALI were associated with PFS in NSCLC patients receiving ICIs (*P* < 0.05); smoking history, and adverse reactions were associated with OS (*P* < 0.1). Results are shown in Table 3 .

**Table 3:** Univariate Analysis Results for PFS and OS in NSCLC Patients Receiving ICIs Treatment

Total evaluated		*n*	PFS			OS		
			mPFS(Months)	χ²/HR(95%CI)	*P* value	mOS(Months)	χ²/HR(95%CI)	*P* value
Gender				0.888	0.346		1.76	0.675
	female	39	12			27		
	male	173	11			25		
Age				7.524	0.006		2.479	0.115
	<60	74	13			31		
	≥ 60	138	10			24		
Smoking status				0.576	0.448		3.27	0.071
	Yes	133	11			21		
	NO	79	12			35		
Histological Type				0.007	0.932		0.719	0.397
	Adenocarcinoma	71	10			29		
	Squamous Cell Carcinoma	141	12			22		
Past Medical History				1.578	0.209		0.017	0.895
	Yes	79	11			24		
	NO	133	12			26		
Tumor staging				0.881	0.348		0.146	0.703
	III	74	13			27		
	IV	138	10			22		
Combined with anti-angiogenic therapy				2.541	0.111		0.12	0.729
	Yes	41	12			31		
	NO	171	11			24		
Treatment line				4.382	0.036		0.131	0.717
	1st	184	11			40		
	2nd and above	28	6			25		
ADR				1.08	0.299		3.502	0.061
	Yes	62	13			31		
	NO	150	10			22		
ALI		212	-	0.97 (0.947,0.995)	0.019	-	0.995 (0.988,1.003)	0.228
PNI		212	-	0.993 (0.988,0.999)	0.013	-	0.981 (0.939,1.024)	0.38

#### Multivariate Cox regression analysis

Factors with *P* < 0.1 in univariate analyses of PFS and OS, as well as clinically meaningful indicators and variables with borderline baseline differences (including tumor stage, treatment line, history of underlying diseases, and combined anti-angiogenic therapy), were entered into a multivariable Cox regression model to adjust for confounding factors.After multivariable adjustment, no significant differences were observed between the two treatment groups in terms of PFS (HR = 1.128, 95% CI 0.596–2.133, *P* = 0.712) or OS (HR = 0.664, 95% CI 0.378–1.165, *P* = 0.153). The occurrence of treatment-related adverse events showed a marginal correlation with improved OS (HR = 0.569, 95% CI 0.295–1.098, *P* = 0.093), but did not reach statistical significance.Additionally, ALI, PNI, combined anti-angiogenic therapy, tumor stage, history of underlying diseases, and treatment line were not identified as independent prognostic factors (all *P* > 0.05), as detailed in Table 4.

**Table 4:** Multivariate Cox regression analysis of factors affecting PFS and OS in patients with NSCLC receiving ICIs treatment

Total evaluated	PFS		OS	
	Pvalue	HR(95%CI)	*P* value	HR(95%CI)
Sintilimab VSTislelizumab	0.712	1.128(0.596–2.133)	0.153	0.664(0.378–1.165)
Age(≧60 or๤60)	0.619	0.853(0.456–1.832)		
Smoking status(YES or NO)			0.139	1.579(0.862–2.892)
Tumor staging(IIIor IV)	0.979	1.008(0.555–1.832)	0.968	1.011(0.588–1.738)
Past Medical History(YES or NO)	0.655	0.866(0.461–1.627)	0.736	1.101(0.631–1.919
Combined with anti -angiogenic therapy(YES or NO)	0.179	1.61(0.804–3.223)	0.56	1.224(0.62–2.418)
Treatment line(1st or 2nd and above)	0.164	0.548(0.235–1.279)	0.846	0.911(0.357–2.324)
ADR(YES or NO)			0.093	0.569(0.295–1.098)
ALI	0.32	0.994(0.983–1.006)	0.385	0.996(0.986–1.005)
PNI	0.26	0.968(0.913–1.025)	0.533	0.983(0.93–1.038)

## Discussion

With the influx of numerous immunotherapy drugs into clinical practice, treatment regimens for NSCLC patients have been continuously optimized. Immunotherapy, represented by PD-1 inhibitors, has emerged as a new anti-tumor treatment modality following traditional therapies and molecularly targeted therapies, profoundly transforming clinical management strategies for NSCLC and continuously improving patient outcomes. Treatment regimens combining immunotherapy with platinum-based double-agent chemotherapy have become the new standard for first- and second-line treatment in NSCLC patients, and the addition of immunotherapy continues to improve clinical outcomes. The results of this study show that the median progression-free survival (mPFS) and median overall survival (mOS) in the tislelizumab and sintilimab groups were 14.0 months and 7.0 months, respectively; the objective response rates (ORR) were 39.52% and 18.18%, respectively; and the disease control rates (DCR) were 66.13% and 70.54%, respectively. According to clinical trial data from existing studies such as ORIENT-11 [9], ORIENT-12 [10], RATIONALE 304 [11], and RATIONALE 307 [12], the median progression-free survival (mPFS) for tislelizumab and sintilimab used as first-line treatment for patients with non-small cell lung cancer (NSCLC) ranges from 5.5 to 9.8 months, median overall survival (mOS) ranges from 21.4 to 26.1 months (OS data in ORIENT-12 were immature at the time of analysis), the overall response rate (ORR) ranges from 51.9% to 74.8%, and the disease control rate (DCR) ranges from 86% to 94%. The RATIONALE-303 study showed that for tislelizumab as second- or third-line treatment in patients with locally advanced or metastatic squamous or non-squamous NSCLC, the median PFS (mPFS) was 4.1 months, and the median overall survival (mOS) was 16.9 months. The ORR was 21.9%–22.6%, and the DCR was 68.7% [13]. It can be seen that the ORR and DCR rates in this study were lower than those in the aforementioned clinical studies; This may be related to the fact that the results of this study come from a real-world retrospective clinical study, which, unlike an RCT, did not involve strict patient enrollment, good nutritional status, timely follow-up, or prompt management of adverse events. Real-world clinical studies involve more complex patient populations than clinical trials, with numerous confounding factors, including differences in treatment adherence, varying economic circumstances, and the presence of related complications or underlying conditions in most patients; Furthermore, prior to receiving PD-1 therapy, more than half of the enrolled patients had not undergone PD-1/PD-L1 expression testing, leaving their PD-1/PD-L1 expression levels unknown. PD-1/PD-L1 is the most extensively studied therapeutic target [14] and has been proven to be an effective biomarker for prognosis and predicting sensitivity to immune checkpoint inhibitors. Numerous studies have confirmed the clinical significance of PD-1/PD-L1 antibodies and their impact on the prognosis of human cancers [15], which explains why the results of real-world clinical studies differ from those of RCTs.

Further analysis of the aforementioned clinical studies [9–12] revealed that median progression-free survival (mPFS) was slightly longer in the tislelizumab group than in the sintilimab group. In this study, the mPFS in the overall population of the tislelizumab group was 14 months, which was superior to the 7 months observed in the overall population of the sintilimab group. The median overall survival (mOS) in the sintilimab group was 24.0 months, slightly shorter than the 27 months observed in the tislelizumab group; however, this difference was not statistically significant (*P* > 0.05). This suggests that while tislelizumab offers greater short-term survival benefits when used as first-line therapy for NSCLC, the two drugs are comparable in terms of long-term survival benefits; Similar to the findings of Chen et al [16, 17], this study was conducted at multiple centers in China and retrospectively enrolled 913 NSCLC patients receiving first-line treatment with PD-1 inhibitors (such as sintilimab or tislelizumab). The results showed that the median PFS (mPFS) was longer in the tislelizumab group than in the sintilimab group, but the difference was not statistically significant (*P* > 0.05). The results of this study suggest that mPFS is longer than that reported in the RATIONALE 307, RATIONALE 304, ORIENT-11, and ORIENT-12 clinical trials. This may be because most patients in the real-world setting received chemotherapy or were concurrently treated with anti-angiogenic agents. While chemotherapy was previously thought to suppress the immune system, recent studies have shown that it not only directly kills cancer cells but also positively modulates the immune system, alters the local tumor immune microenvironment, and prolongs the efficacy of immunotherapy [18]. Currently, combination therapy has become an effective approach to amplify the benefits of PD-1/PD-L1 immunotherapy and overcome or delay drug resistance. Some patients with advanced NSCLC can survive for 5 years or longer after receiving PD-1 inhibitor treatment, thereby improving PFS. Second, both Tislelizumab and Sintilimab are domestically produced anti-PD-1 IgG4 monoclonal antibodies approved for first-line treatment of advanced NSCLC. The core structural difference lies in Fc region modification: Tislelizumab is an Fc-engineered variant that eliminates FcγR binding, theoretically reducing T-cell exhaustion caused by ADCP/ADCC and enhancing antitumor immunity; Sintilimab, on the other hand, possesses a wild-type IgG4 Fc. Although Tislelizumab has a theoretical advantage in T-cell protection, this real-world study, after adjustment for multiple factors, did not observe significant differences in PFS or OS between the two groups. This may be related to balanced baseline characteristics, consistent chemotherapy regimens, and comparable efficacy of PD-1 pathway inhibition in clinical practice. It should be noted that since sintilimab was approved for marketing in China earlier than tislelizumab, the follow-up duration in the sintilimab group was significantly longer than that in the tislelizumab group. Furthermore, differences in the approved indications for lung cancer and the number of treatment lines between the two drugs may have led to variations in the duration of treatment among enrolled patients and may be related to subsequent treatment responses. Consequently, at the time of follow-up cutoff, data for some patients in both groups who had not yet progressed or were still alive were subject to missing-at-censoring treatment, which may have led to an underestimation of PFS and OS in both groups.

This study found a marginal positive association between the occurrence of treatment-related adverse events and overall survival benefits in patients; however, this association did not reach statistical significance. Numerous studies have shown that the occurrence of immunotherapy-related adverse events, such as immune-related endocrine toxicity, is associated with a favorable clinical prognosis in patients with malignant tumors [19]. Most studies confirm that the presence of moderate immune-related adverse events often indicates that the body’s antitumor immune response has been effectively activated, leading to a better long-term survival prognosis. This trend did not reach statistical significance in the present study. This may be due to the fact that, as a retrospective study, the severity of adverse events was not sufficiently emphasized, leading to some being overlooked. Additionally, no stratification analysis was performed based on the severity of adverse events, preventing differentiation of their varying impacts on prognosis. Furthermore, clinical management of symptomatic supportive care following the onset of adverse events was inconsistent, which may have confounded the final survival outcomes. Third, as a non-single-center study, this research was limited by sample size and insufficient follow-up duration, which reduced the statistical power to detect this weak association. Therefore, these results require validation through future large-scale, multicenter clinical studies. At the same time, this suggests that clinicians should prioritize the stratified management of adverse reactions, maintaining an effective antitumor immune state within safe and controllable limits, which may lead to improved prognostic outcomes.

Cancer is a chronic inflammatory process. Systemic inflammation plays a critical role in tumor survival, proliferation, angiogenesis, immunosuppression, and catabolism. PD-L1 expression levels are currently the only biomarker approved by the FDA for predicting response to immune checkpoint inhibitors [20]; however, its clinical adoption has been limited by challenges such as difficulty in obtaining samples and high testing costs, and some PD-L1-positive patients still exhibit poor response to immunotherapy [21]. There is an urgent clinical need to identify new biomarkers to predict the response to ICI therapy in patients with advanced non-small cell lung cancer (NSCLC). ALI, a novel prognostic biomarker for NSCLC patients, is composed of body mass index (BMI), albumin (ALb), and the neutrophil-to-lymphocyte ratio (NLR). In lung cancer patients with low ALI—defined by low BMI, low ALb, and high NLR—overall survival (OS), progression-free survival (PFS), and recurrence-free survival are shorter [22, 23]. ALI is an independent risk factor for patients, and a low ALI may be associated with adverse outcomes such as shorter PFS and early disease progression [24, 25]. Current studies both domestically and internationally indicate that ALI is significantly associated with survival in NSCLC patients and can effectively predict the prognosis of different treatment modalities for NSCLC patients, holding significant clinical importance; However, the results of this study, after adjustment for multiple confounding factors, indicate that ALI is not a prognostic factor for patients. First, the study population consisted entirely of patients with advanced (Stage III and IV) lung cancer, whose overall nutritional status and systemic inflammation levels were relatively homogeneous, resulting in minimal individual variation in ALI values, making it difficult to effectively distinguish between subgroups with different prognoses; Second, due to immune deficiency, malnutrition, psychological stress, and treatment-related adverse events (such as bone marrow suppression), patients with advanced lung cancer are more susceptible to infections, and their risk of dying from life-threatening infections is far higher than that of non-cancer patients [26]. factors such as comorbidities, symptomatic supportive interventions during treatment, and dynamic fluctuations in the body’s inflammatory state—combined with the fact that most lung cancer patients receive concurrent antibiotic therapy during anticancer treatment—all interfere with the predictive validity of baseline ALI for long-term survival. Studies have shown that the use of antibiotics before or during ICI therapy can shorten mOS by more than 6 months, and the impact on patient mOS is even greater when patients take antibiotics within 60 days before or after the start of ICI therapy [27, 28]. The ALI index may have greater prognostic value in populations with significant differences in performance status and in those receiving monotherapy. Future stratified studies could be conducted to further clarify the applicability and optimal cutoff values of ALI in patients receiving PD-1 inhibitors combined with chemotherapy.

This study has certain limitations: (1) It collected data from a single center only and had a small sample size; therefore, the findings require validation through additional multicenter, large-scale prospective studies; (2) This study is a retrospective analysis based on real-world data. While it accurately reflects clinical practice, it may be subject to data recording biases and cannot control for confounding factors through methods such as randomization or matching, thereby posing a risk of bias in the results; (3) Since PD-L1 testing was not performed in some of the included patients, and test results were missing in others due to the retrospective nature of the study, it was not possible to compare the relationship between high and low PD-L1 expression and factors influencing PFS and OS; (4) Due to limitations in the duration of follow-up, further long-term follow-up is required for the OS and PFS data to provide a more detailed analysis of patient survival outcomes.

## Conclusions

In summary, compared with the sintilimab group, the combination of tislelizumab and chemotherapy may improve the objective response rate and prolong progression-free survival in the short term for patients with advanced non-small cell lung cancer; however, in terms of long-term survival benefits, sintilimab and tislelizumab combined with chemotherapy demonstrate comparable efficacy in patients with advanced NSCLC.

## Data Availability

The data were available from the corresponding author on reasonable request.

## References

[1] Riely GJ, Wood DE, Ettinger DS et al. Non-small cell lung cancer, version 4.2024, NCCN clinical practice guidelines in oncology [J].J Natl Compr Canc Netw, 2024,22(4): 249–74. 10.6004/jnccn.2204.0023 38754467

[2] Siegel RL, Giaquinto AN, Jemal A. Cancer statistics,2024 [J]. CA Cancer J Clin. 2024;74(1):12–49. 10.3322/caac.21820. .Epub 2024 Jan 17. 38230766

[3] De Castro G Jr, Kudaba I, Wu YL, et al. Five-year out-comes with pembrolizumab versus chemotherapy as first-line therapy in patients with non-small-cell lung cancer and programmed death ligand-1 tumor proportion score ≥ 1% in the KEYNOTE-042 study [. J] J Clin Oncol. 2023;41(11):1986–91. 10.1200/JCO.21.02885. PMC10082298 36306479

[4] Shiraishi Y, Nomura S, Sugawara S, et al. Comparison of platinum combination chemotherapy plus pembrolizumab versus platinum combination chemotherapy plus nivolumab ipilimumab for treatment-naive advanced non-small-cell lung cancer in Japan (JCOG2007): an open-label,multicentre, randomised, phase 3 trial [. J] Lancet Respir Med. 2024;12(11):877–87. 10.1016/S2213-2600(24)00185-1. 39159638

[5] Chinese Society of Clinical Oncology (CSCO) Guidelines Working Committee. Chinese Society of Clinical Oncology (CSCO) guidelines for the diagnosis and treatment of non-small cell lung cancer: 2025 edition. 2025.

[6] Zheng X, l;Wang X, h;Sun Y. n Analysis of clinical efficacy of first-line immunotherapy combined with radiotherapy in patients with brain metastasis of non-small cell lung cancer[J]. Chin J Radiation Oncol 2023,32(12):1044–50. 10.3760/cma.j.cn113030-20221009 -00333.

[7] Lahiri A, Maji A, Potdar PD et al. Lung cancer immunotherapy:progress,pitfalls,and promises[J].Mol Cancer,2023,22(1):40. 10.1186/s12943-023-01740-y PMC9942077 36810079

[8] Qian J-Y, Wu L-L, Zhang L-Y, et al. Prognostic evaluation of stage I lung adenocarcinoma based on systematic inflammatory response[J]. JNCI Cancer Spectr. 2023;7(6):pkad090. 10.1093/jncics/pkad090. PMC10660118 37941434

[9] Yang Y, Wang Z, Fang J et al. Efficacy and Safety of Sintilimab Plus Pemetrexed and Platinum as First-Line Treatment for Locally Advanced or Metastatic Nonsquamous NSCLC:a Randomized, Double-Blind, Phase 3 Study (Oncology pRogram by InnovENT anti-PD-1-11)[J].J Thorac Oncol,2020,15(10): 1636–46. 10.1016/j.jtho.2020.07.014 32781263

[10] Zhang L, Wang Z,Fang J et al. Final overall survival data of sintilimab plus pemetrexed and platinum as First-Line treatment for locally advanced or metastatic nonsquamous NSCLC in the Phase 3 ORIENT-11 study[J].Lung Cancer,2022,171: 56–60. 10.1016/j.lungcan.2022.07.013 35917647

[11] Lu S, Wang J, Yu Y et al. Tislelizumab plus chemotherapy as first-line treatment of locally advanced or metastatic nonsquamous non-small-cell lung cancer(final analysis of RATIONALE-304:a randomized phase Ⅲ trial)[J].ESMO Open,2024, 9(10):103728. 10.1016/j.esmoop.2024.103728 PMC11549519 39461773

[12] Wang J, Lu S, Yu X et al. Tislelizumab plus chemotherapy versus chemotherapy alone as first-line treatment for advanced squamous non-small-cell lung cancer:final analysis of the randomized,phase Ⅲ RATIONALE-307 trial[J].ESMO Open,2024, 9(10):103727. 10.1016/j.esmoop.2024.103727 PMC11549530 39461775

[13] Zhou CC, Huang D et al. Tislelizumab versus docetaxel in patients with previously treated advanced NSCLC(RATIONALE-303):a phase 3,open-label,randomized controlled trial[J]. J Thorac Oncol 2023,18(1):93–105. 10.1016/j.jtho.2022.09.217 36184068

[14] Mok TSK, Wu YL, Kudaba I et al. Pembrolizumab versus chemotherapy for previously untreated, PD-L1-expressing, locally advanced or metastatic non-small-cell lung cancer(KEYNOTE-042):a randomised, open-label, controlled, phase 3 trial [J].Lancet (London,England),2019,393(10183): 1819–30. 10.1016/S0140-6736(18)32409-7 30955977

[15] Tang Q, Chen Y, Li X et al. The role of PD-1/PD-L1 and application of immune-checkpoint inhibitors in human cancers [J].Frontiers in immunology,2022,13: 964442. 10.3389/fimmu. 2022.964442. PMC9513184 36177034

[16] Chen S, Li T, Yang W et al. Comparative efficacy of six programmed cell death Protein-1 inhibitors as first-line treatment for advanced non-small cell lung cancer: a multicenter retrospective cohort study[J].Front Pharmacol,2024,15:1390872DOI 10.3389/fphar.2024.1390872 PMC11148465 38835662

[17] Shi Y, Wu L, Yu X et al. Sintilimab versus docetaxel as second-line treatment in advanced or metastatic squamous non-small-cell lung cancer: an open-label, randomized controlled phase 3 trial (ORIENT-3) [J].Cancer communications (London, England),2022,42(12):1314–30. 10.1002/ cac2.12385 10.1002/cac2.12385 PMC9759762 36336841

[18] Galluzzi L, Buqué A, Kepp O et al. Immunogenic cell death in cancer and infectious disease [J]. Nat reviews Immunol 2017,17(2):97–111. 10.1038/nri.2016.107 27748397

[19] WuL. XuY,WangX,etal.Thyroid dysfunction after immunecheckpoint inhibitor treatment in a single-center Chinese cohort:a retrospective study[J].Endocrine,2023,81(1):123–33. 10.1007/ s12020-023-03323-9 10.1007/s12020-023-03323-9 36867366

[20] Uruga H, Mino-Kenudson M. Predictive biomarkers for response to immune checkpoint inhibitors in lung cancer:PD-L1 and beyond[J].Virchows Arch,2021,478(1):31–44. 10.1007/s00428-021- 03030–8. 33486574

[21] Rizzo A. Identifying optimal first -line treatment for advanced non -small cell lung carcinoma with high PD-L1 expression: a matter of debate [J]. Br J Cancer. 2022;127(8):1381–2. 10. 1038/s41416-022-01929-w. 10.1038/s41416-022-01929-w PMC9553978 36064585

[22] Zhang Y, Chen B. Prognostic Value of the Advanced Lung Cancer Inflammation Index in Patients with Lung Cancer:A Meta-Analysis[J].Dis Markers,2019:2513026. 10.1155/2019/2513026 PMC6636448 31354887

[23] Wang Y, Lin L,Ji Y et al. Prognostic value of the advanced lung cancer inflammation index in early-stage non-small cell lung cancer patients undergoing video-assisted thoracoscopic pulmonary resection[J]. Ann Palliat Med 2020,9(3):721–9. 10.21037/apm.2020.03.18 32312059

[24] Tomita M, Ayabe T, Maeda R et al. Comparison of Inflammation-Based Prognostic Scores in Patients undergoing Curative Resection for Non-small Cell Lung Cancer[J]. World J Oncol 2018,9(3):85–90. 10.14740/wjon1097w PMC6031234 29988766

[25] Shiroyama T, Suzuki H,Tamiya M et al. Pretreatment advanced lung cancer inflammation index(ALI)for predicting early progression in nivolumab-treated patients with advanced non-small cell lung cancer[J]. Cancer Med 2018,7(1):13–20. 10.1002/cam4.1234 PMC5773945 29150906

[26] Zheng Y, Chen Y, Yu K, et al. Fatal Infections Among Cancer Patients: A Population-Based Study in the United States[J]. Infect Dis Ther. 2021;10(2):871–95. 10.1007/s40121-021-00433-7. PMC8116465 33761114

[27] Alkan Şen G, Nihan Ş, Ö,DEĞERLI, E et al. Effect of antibiotic treatment on immune checkpoint inhibitors efficacy in patients with advanced non-small cell lung cancer[J].Lung Cancer,2023,184:107347. 10.1016/j.lungcan.2023.107347 37597304

[28] Wu Q, Liu JJ, Wu SM, et al. The impact of antibiotics on efficacy of immune checkpoint inhibitors in malignancies: a study based on 44 cohorts. Int Immunopharmacol. 2021;92:107303. 10.1016/j.intimp.2020.107303. 33465728

